# Long-Range Memory in Literary Texts: On the Universal Clustering of the Rare Words

**DOI:** 10.1371/journal.pone.0164658

**Published:** 2016-11-28

**Authors:** Kumiko Tanaka-Ishii, Armin Bunde

**Affiliations:** 1 The University of Tokyo, Research Center for Advanced Science and Technology, Tokyo, 153-8904, Japan; 2 Universität Giessen,Institut für Theoretische Physik,Giessen,35392, Germany; University of Warwick, UNITED KINGDOM

## Abstract

A fundamental problem in linguistics is how literary texts can be quantified mathematically. It is well known that the frequency of a (rare) word in a text is roughly inverse proportional to its rank (Zipf’s law). Here we address the complementary question, if also the rhythm of the text, characterized by the *arrangement* of the rare words in the text, can be quantified mathematically in a similar basic way. To this end, we consider representative classic single-authored texts from England/Ireland, France, Germany, China, and Japan. In each text, we classify each word by its rank. We focus on the *rare* words with ranks above some threshold *Q* and study the lengths of the (return) intervals between them. We find that for *all* texts considered, the probability *S*_*Q*_(*r*) that the length of an interval exceeds *r*, follows a perfect Weibull-function, *S*_*Q*_(*r*) = exp(−*b*(*β*)*r*^*β*^), with *β* around 0.7. The return intervals themselves are arranged in a long-range correlated self-similar fashion, where the autocorrelation function *C*_*Q*_(*s*) of the intervals follows a power law, *C*_*Q*_(*s*) ∼ *s*^−*γ*^, with an exponent *γ* between 0.14 and 0.48. We show that these features lead to a pronounced clustering of the rare words in the text.

## Introduction

Can literature be characterized by mathematical laws? According to Zipf [1], the frequency of a word as function of its rank follows approximately a power law, and also the number of different words in a text increases with its length roughly by a power law [2, 3]. The question is if also the rhythm of the text characterized by the arrangement of lower and higher ranked words, can be quantified mathematically in a similar basic way. In the last decades, when analyzing the rhythm of a text, the text was usually mapped onto a sequence {*y*_*i*_}, *i* = 1, …, *N*, of numbers that specify either the lengths of words or sentences, or the ranks or frequencies of each word, or mapped into various binary sequences that specify the occurrences of specific words. Then record analysis methods from statistical physics like Hurst analysis [4], (multifractal) detrended fluctuation analysis (DFA and MF-DFA) [5, 6], or entropy measures have been used to search for linear and nonlinear memory in the text [7–13].

For example, Ebeling and Neimann [8] transformed the letters in the Bible, Grimm Tales, and Moby Dick into binary sequences of appearance/non-appearance and used DFA and power-spectrum analysis to detect correlations. Montemurro and Pury [9] applied Hurst analysis to rank transformed texts (Shakespeare, Dickens, Darwin collections) while Kosmidis et al. [10] applied DFA to the frequencies of the words. All authors found that the studied fluctuation functions for the considered texts were significantly different from shuffled texts, suggesting Hurst exponents well above 1/2 and thus indicating long-term memory in the texts. It has been argued by Altmann et al. [11] how the correlations could flow from highly structured linguistic levels down to the building blocks of a text (words, letters, etc.). Moreover, Altmann et al. [12] considered USENET discussion groups and indicated that the cumulative distribution of the intervals between specific words follow a Weibull function. They emphasized that different values of the exponent may correspond to different semantic categories. But despite all efforts, the *specific* mathematical laws that govern the rhythm of a text remained unclear. As we point out here, one of the reasons for this limitation lies in the large amount of white noise which, in addition to the long-range memory, characterizes the arrangement of words in a text and prohibits showing the degree of memory in the common Hurst or DFA analysis.

In this article, we apply the return-interval technique (also called peak-over-threshold method) to single-authored texts, for analyzing the arrangement of the rare words in the text. The method itself has been rigorously established in the statistical physics domain, and has been effective in analyzing extremes in natural and financial sciences (see, e.g., [14–21]). When applying to language data, the return-interval technique has the great advantage by not requiring any mapping of the words to numbers.

## Materials and Methods

In the return-interval analysis of extreme events one considers, in records with *N* data points, the *N*_*Q*_ rarest events and investigates the statistics of the intervals between consecutive events. By definition, *N*_*Q*_/*N* is the fraction of rare events, and *R*_*Q*_ = *N*/*N*_*Q*_ is the mean length of the intervals.

Accordingly, in a text with *N* words, we consider the *fraction N_Q_/N of the rarest words* that by definition have a rank above *Q*. Two consecutive rare words are separated by *l* non-rare words, and the (return) interval between them is *r* = *l* + 1. As in studies of catastrophic rare events, we focus on the statistics and the arrangement of these return intervals for fixed *Q* and how it changes when *Q* is increased. The mean interval length *R*_*Q*_ represents the characteristic length scale. Since the power law relation between rank and frequency of a word observed by Zipf is not strictly universal and changes in different texts [22, 23], *R*_*Q*_ is not a universal function of *Q* (see Fig B in S1 File). In the following, for comparing different texts, instead of keeping *Q* fixed, we keep *R*_*Q*_ = *N*/*N*_*Q*_ fixed. We like to note that our study complements and extends a previous study by Altmann et al. [12] where exclusively the return intervals of a specific word (that occurs *N*_*s*_ times in the text) have been considered. The mean distance characterizing this word is accordingly *N*_*s*_/*N*, which has been coined wavelength by Zipf [1]. In contrast, *R*_*Q*_ considered here is the mean distance between all rare words with rank above *Q*.

In our study, we have analyzed the following 10 texts: (i) Les Miserables by V. Hugo (French), number of words *N* = 691407, maximum rank *Q*_max_ = 31659, (ii) Ulysses by J. Joyce (English), *N* = 325692, *Q*_max_ = 34359, (iii) Phänomenologie des Geistes by G. Hegel (German), *N* = 220159, *Q*_max_ = 9866, (iv) Hong Lou Meng by C. Xueqin (Chinese), *N* = 703033, *Q*_max_ = 18311, (v) Magura by K. Yumeno (Japanese), *N* = 273928, *Q*_max_ = 15883, (vi) Essai by M. Montaigne (French), *N* = 822630, *Q*_max_ = 41235, (vii) The Great Boer War by A.C. Doyle (English), *N* = 249384, *Q*_max_ = 13408, (viii) Die Traumdeutung by S. Freud (German), *N* = 250564, *Q*_max_ = 28864, (ix) Journey to the West by C. Wu (Chinese), *N* = 649217, *Q*_max_ = 14061, and (x) Daibosatsu Toge by K. Nakazato (Japanese), *N* = 2951319, *Q*_max_ = 49099. The Chinese and Japanese texts were preprocessed into words with the ICTCLAS and MeCab, respectively, which are standard software packages for chunking.

**Fig 1:**
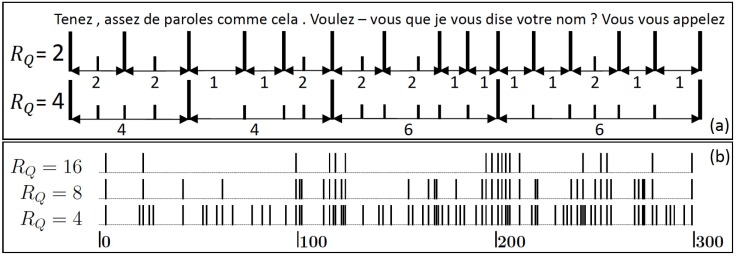
Return intervals in a text. (a) shows the word sequence of Les Miserables from word 31096 to word 31116. Punctuations are considered as words. The sequences beneath illustrate how the return intervals between rare words and their lengths are defined: For *R*_*Q*_ = 2 and 4, only words with ranks above the corresponding *Q* value (here: *Q* = 46 and 544, respectively) are picked out and denoted by the large bars. The other words are denoted by the small bars. The return intervals are the intervals between consecutive large bars, i.e. the number of small bars between 2 consecutive large bars plus 1, and are listed beneath the sequences. (b) shows, in a segment of 300 words, the position of words with ranks above *Q* = 544, 2731, and 7265. The corresponding mean return times are *R*_*Q*_ = 4, 8, and 16, respectively. For *R*_*Q*_ = 8 and 16, the words are not distributed homogeneously but tend to cluster.

Fig 1a illustrates the intervals for a certain sequence in Les Miserables, for *R*_*Q*_ = 2 and 4. Words with ranks above *Q* are denoted by large bars, otherwise by short bars. Fig 1b shows a larger sequence, for *R*_*Q*_ = 4, 8, and 16. The bars are for words above the respective *Q* values. The intervals between them characterize the rhythm of the text. One can see by eye that the bars, in particular for *R*_*Q*_ = 16, are not homogeneously distributed, but tend to cluster. This means, short intervals have a tendency to follow short intervals, while long intervals have a tendency to follow long intervals.

## Results

### Exceedance Probability

For analyzing the statistics of the intervals, for fixed *R*_*Q*_, and discovering the mathematical laws behind them, we have determined (i) how often an interval of length *r*, *r* = 1, 2, 3, …, appears in a text, and (ii) how often intervals above a certain length *r* appear. After division by the total number of intervals *N*_*Q*_ − 1, (i) yields the probability distribution *P*_*Q*_(*r*) of the interval length, while (ii) yields the exceeding probability *S*_*Q*_(*r*). *S*_*Q*_(*r*) is the probability that in a text an interval between consecutive words with rank above *Q*, is longer than a given interval length *r*. By definition, *S*_*Q*_(0) = 1 and *S*_*Q*_(*r* − 1) − *S*_*Q*_(*r*) = *P*_*Q*_(*r*) for *r* ≥ 1.

**Fig 2:**
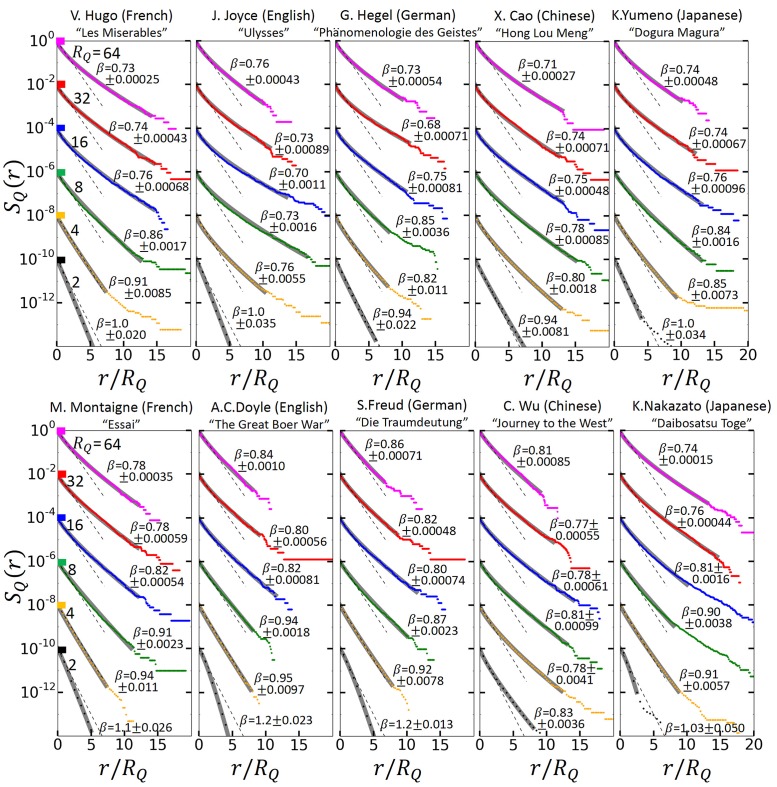
The probability *S*_*Q*_(*r*) that in a text the return intervals between words with rank above *Q* (see Fig 1) exceed a certain length *r*. We consider *Q* values where the mean return intervals have lengths *R*_*Q*_ = 64, 32, 16, 8, 4, and 2 (from top to bottom). By definition, *S*_*Q*_(0) = 1. For transparency, we have multiplied *S*_*Q*_ for *R*_*Q*_ = 32, 16, 8, 4, and 2 by 10^−2^, 10^−4^, 10^−6^, 10^−8^, and 10^−10^, respectively, and plotted *S*_*Q*_ as a function of *r*/*R*_*Q*_. The dots are the numerical results. The gray lines are the best fit to *S* = exp[−*b*(*r*/*R*_*Q*_)^*β*^], with $b={\left[\int_{0}^{\infty }dx\exp\left(-x^{\beta }\right)\right]}^{\beta }$ for *R*_*Q*_ ≥ 16 (see SI). The value of *β* is shown for each fit with its error bar as the standard deviation of the fit. The figure shows that for all texts and *R*_*Q*_ above 2, stretched exponentials (where *β* < 1) make a remarkable fit. In each text, approximately the same exponent *β* characterizes *S*_*Q*_ for *R*_*Q*_ ≥ 16. The exponent varies only slightly in the different texts: Means and standard deviations were 1.1 and 0.13 for *R*_*Q*_ = 2, 0.86 and 0.067 for *R*_*Q*_ = 4, 0.85 and 0.059 for *R*_*Q*_ = 8, 0.77 and 0.037 for *R*_*Q*_ = 16, 0.76 and 0.037 for *R*_*Q*_ = 32, 0.77 and 0.048 for *R*_*Q*_ = 64. The dashed straight lines are for the shuffled texts. For 20 shuffled texts of Les Miserables, the means were 1.0 for all *R*_*Q*_s, with standard deviations of 0.0052, 0.013, 0.0080, 0.012, 0.010, 0.028, for *R*_*Q*_ = 2, 4, 8, 16, 32, and 64, respectively.

Fig 2 shows *S*_*Q*_(*r*), for the 10 texts considered, for *R*_*Q*_ = 2, 4, 8, 16, 32 and 64. The dashed lines show *S*_*Q*_ for the shuffled texts. It is easy to show that in this case, *S*_*Q*_(*r*) = (1 − 1/*R*_*Q*_)^*r*^ ≡ exp(−|ln(1 − 1/*R*_*Q*_)|*r*), yielding *S*_*Q*_(*r*) ≅ exp(−*r*/*R*_*Q*_) for *R*_*Q*_ ≫ 1. Accordingly, deviations from a simple exponential can be viewed as measure of the complexity of a text. The figures show that for *R*_*Q*_ = 2, i.e. when half of the total words (with ranks above the median rank) are considered, *S*_*Q*_ is described, for most texts, by a simple exponential. This changes when we increase *R*_*Q*_. For *R*_*Q*_ ≥ 4, in all texts *S*_*Q*_(*r*) follows a perfect *“stretched”* exponential

$$S_{Q}\left(r\right)=\exp\left(-b\left(\beta \right){\left(r/R_{Q}\right)}^{\beta }\right),$$ (1)

where the exponent *β* first slightly decreases with increasing *R*_*Q*_. For *R*_*Q*_ above 4, *β* is between 0.71 and 0.86. The parameter *b* depends on *β*. We show in the SI that for large *R*_*Q*_, $b={\left[\int_{0}^{\infty }dx\exp\left(-x^{\beta }\right)\right]}^{\beta }$, which indeed gave the best fit in all texts for *R*_*Q*_ ≥ 16. Stretched exponential functions, sometimes also referred to as Weibull functions, appear in science in many contexts, in materials science [24] as well as in climate and earth sciences [16–19], just to mention a few. In our case, the agreement between the measured data and the stretched exponential form is exceptionally good. We like to note that our result also supports the previous findings in [12] where the return intervals between a certain single word in a text have been analyzed and for the corresponding exceedance probabilities also Weibull functions have been considered.

### Clustering of rare words

The knowledge of *S*_*Q*_(*r*) allows us to quantify the clustering of the rare words (with rank above *Q*) noticed in Fig 1. Let us assume that after a rare word at a certain position in the text, the following *t* words have ranks below *Q*. The question we ask is: What is the probability *W*_*Q*_(*t*, Δ*t*) that there is at least one word with rank above *Q* among the next Δ*t* words at positions *t* + 1, *t* + 2, ⋯, *t* + Δ*t* after the considered rare word. In the theory of extreme events, *W* is of great importance. It gives the probability that an extreme event will happen in the next Δ*t* time steps, provided that the last extreme event occurred *t* time steps ago. It can be easily verified that this probability (which is also called “hazard function”), is related to the exceedance probability *S*_*Q*_(*r*) by

$$W_{Q}\left(t,\Delta t\right)=\frac{S_{Q}\left(t\right)-S_{Q}\left(t+\Delta t\right)}{S_{Q}\left(t\right)}.$$ (2)

The nominator is the probability that a rare word occurs at positions between *t* and *t* + Δ*t*. The denominator is a normalization factor ensuring *W*_*Q*_(*t*, ∞) = 1, this way taking into account the condition that there were no rare words at the *t* positions after the considered rare word.

Combining Eq (2) with Eq (1) yields

$$W_{Q}\left(t,\Delta t\right)=1-\exp\left(-b\left(\beta \right){\left[\left(t+\Delta t\right)/R_{Q}\right]}^{\beta }\right)/\exp\left(-b\left(\beta \right){\left[t/R_{Q}\right]}^{\beta }\right).$$ (3)

For *t* = 0, Eq (3) reduces to

$$W_{Q}\left(0,\Delta t\right)=1-\exp\left(-b\left(\beta \right){\left[\Delta t/R_{Q}\right]}^{\beta }\right).$$ (4)

For a purely random arrangement of rare words, *β* = 1 and Eq (4) yields $W_{Q}\left(0,\Delta t\right)\equiv W_{Q}^{\left(0\right)}=1-\exp\left(-\Delta t\right)/R_{Q})$ Since *β* in Eq (4) is below 1, *W*_*Q*_(0, Δ*t*) is larger than $W_{Q}^{\left(0\right)}$, i.e. the rare words cluster. As an example, consider Δ*t*/*R*_*Q*_ = 1/64, i.e. we ask what is the probability that directly after a rare word with return period 64 another rare word appears in the text. For a pure random arrangement we have $W_{Q}\;≅\;1/64$, while for a text characterized by *β* = 3/4 we have $W_{Q}\;≅\;1/20$.

### Long-range memory in the return intervals

Next we consider the intrinsic reason for this clustering. We denote the lengths of the consecutive intervals in the text, for fixed *Q* resp *N*_*Q*_, by *r*_*i*_, *i* = 1, 2, …, *L*_*Q*_ = *N*_*Q*_ − 1 and ask, if interval *i* with length *r*_*i*_ and interval *i* + *s* with length *r*_*i* + *s*_ are correlated. To this end, we study the autocorrelation function

$$C_{Q}\left(s\right)=\frac{1/\left(L_{Q}-s\right)\sum_{i=1}^{L_{Q}-s}\left(r_{i}-R_{Q}\right)\left(r_{i+s}-R_{Q}\right)}{1/L_{Q}\sum_{i=1}^{L_{Q}}{\left(r_{i}-R_{Q}\right)}^{2}}.$$ (5)

By definition, *C*_*Q*_(0) = 1. For randomly arranged words (for example, after shuffling the text or the intervals), *C*_*Q*_(*s*) fluctuates around zero for *s* ≥ 1 (see Fig C in S1 File). If there is short-range memory in the intervals, *C*_*Q*_(*s*) will decay exponentially, while in the presence of long-range memory, *C*_*Q*_(*s*) will decay by a power law.

**Fig 3:**
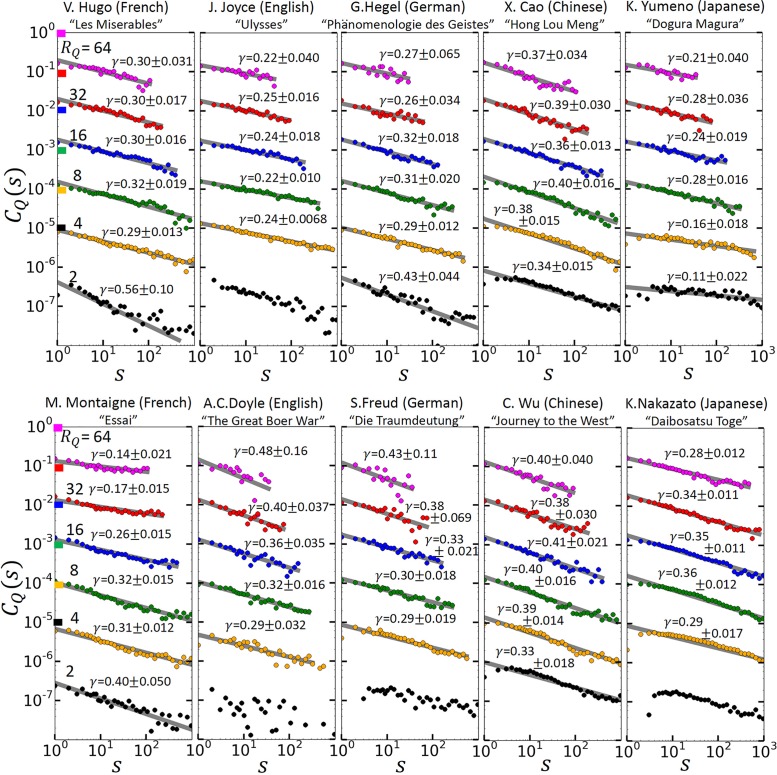
Long-range memory in the rhythm of a text. The figure shows the autocorrelation function *C*_*Q*_(*s*) that quantifies the correlations between the return intervals, for the same *R*_*Q*_ values and the same texts as in Fig 2. For transparency, we have multiplied *C*_*Q*_ for *R*_*Q*_ = 16, 8, 4, and 2 by 10^−1^, 10^−2^, 10^−3^, and 10^−4^, respectively. Since autocorrelation functions are known to show strong finite-size effects [25], we considered only *s*-values up to (*N*_*Q*_ − 1)/100. For *s* above 10, the data were binned logarithmically. The straight lines are the best linear fit to the data, provided all data were positive. The fitted values *γ* are shown with their error bars as standard deviations. At *R*_*Q*_ = 2, the first data point was negative for Ulysses, The Great Boer War, Die Traumdeutung, and Daibosatsu Toge. At *R*_*Q*_ ≥ 4 all texts show clear power-law correlations.

Fig 3 shows, for the same texts and *R*_*Q*_ values as in Fig 2, the autocorrelation function *C*_*Q*_(*s*) *of the return intervals*. In all texts, *C*_*Q*_(*s*) follows, over several decades, a clear power law,

$$C_{Q}\left(s\right)=C_{Q}\left(1\right)s^{-\gamma },s>0.$$ (6)

Accordingly, the intervals are arranged in a self-similar long-range correlated fashion. The exponent *γ* measures how fast the long-range memory decays. There is no clear picture for the behavior of *γ*. In the first 5 texts, for *R*_*Q*_ above 4, *γ* seems to be rather independent of *R*_*Q*_, varying between *γ* = 0.24 for Ulysses and *γ* = 0.38 for Hong Lou Meng. In the second set of texts, *γ* only seems to be independent of *R*_*Q*_ for the Chinese and Japanese texts. The means and standard deviations of *γ* across the 10 texts were 0.36 and 0.036 for *R*_*Q*_ = 2, 0.31 and 0.040 for *R*_*Q*_ = 4, 0.34 and 0.035 for *R*_*Q*_ = 8, 0.34 and 0.049 for *R*_*Q*_ = 16, 0.33 and 0.084 for *R*_*Q*_ = 32, 0.35 and 0.12 for *R*_*Q*_ = 64. For the English and the German text, *γ* increases with *R*_*Q*_, while it decreases for the French text. The long-range memory is the reason for the clustering of the rare words observed in Fig 1, since due to the memory short intervals have the tendency to follow short intervals, and long intervals long ones. We like to note that in purely long-range correlated records, the exponents *β* and *γ* are approximately the same [16, 26] which is not the case here. Also, the exponent *γ* does not depend on *R*_*Q*_ for large *R*_*Q*_. Accordingly, literary texts have a more complex structure than purely long-term persistent records. As we show below, the return intervals contain also a large fraction of white noise, which effectively diminishes the long-term correlations, this way leading to a larger value of *β*.

### Fraction of White Noise

The prefactor *C*_*Q*_(1) characterizes the strength of the long-range memory. For *R*_*Q*_ above 4, *C*_*Q*_(1) is well above 0.1 and approximately text independent (see Table A in S1 File). For records with purely random long-range correlations, one has [25]

$$C_{Q}\left(1\right)\equiv C_{Q}^{\left(0\right)}\left(1\right)≅\left(1-\gamma \right)\left(1-\frac{\gamma }{2}\right).$$ (7)

Since *C*_*Q*_(1) obtained for the 10 texts is below $C_{Q}^{\left(0\right)}\left(1\right)$, white noise is superposed to the long-range correlations.

Accordingly, for each threshold *Q*, the return intervals *r*_*i*_ are a superposition of white noise *η*_wn_(*i*) and long-range memory *η*_lrm_(*i*),

$$r_{i}=a\eta_{\text{wn}}\left(i\right)+\left(1-a\right)\eta_{\text{lrm}}\left(i\right).$$ (8)

Following [25], the fraction of whitenoise *a* can be estimated by

$$a=\frac{1}{1+\sqrt{C_{Q}\left(1\right)/\left[C_{Q}^{\left(0\right)}\left(1\right)-C_{Q}\left(1\right)\right]}}.$$ (9)

We find that for all texts, *a* decreases initially with increasing *R*_*Q*_. For *R*_*Q*_ between 8 and 64, *a* is approximately constant for each text varying between 0.55 (Hong Lou Meng) and 0.69 (Montaigne) (see Table A in S1 File). Accordingly, the fraction of white noise in the return intervals is larger than the fraction of long-range correlated noise. But nevertheless, it is this small fraction with long-range memory that leads to the clustering of the rare events.

### Conditional mean return intervals

**Fig 4:**
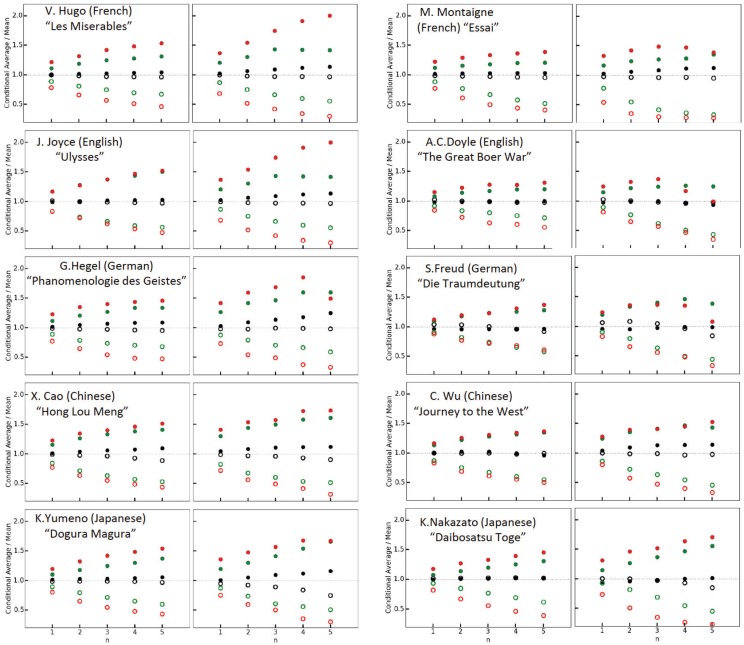
Quantification of the memory effect for 10 texts. For each text, the left-hand graphs show the (conditional) average length of a return interval in units of the mean interval length *R*_*Q*_, for *R*_*Q*_ = 2, 8 and 32, after *n* consecutive short (open circles, below the median) or long (full circles, above the median) intervals. The red, green, and black circles are for *R*_*Q*_ = 32, 8, and 2, respectively. The figure shows that short (long) intervals are more likely followed by short (long) intervals, and quantifies the clustering of rare words for large *R*_*Q*_ that we observed in Fig 1b. When the text is shuffled, all symbols are very close to 1. In the right-hand graphs, the ranked intervals are divided in quarters. Now the short intervals are from the first quarter, the large intervals from the fourth quarter.

To further quantify the clustering of the rare events, we follow [27] and rank, for fixed *R*_*Q*_, the *N*_*Q*_ − 1 intervals according to their length. Then we distinguish between intervals below the median (*short* intervals) and above the median (*long* intervals), and determine the mean interval length after a period of *n* consecutive short resp. long intervals. For each of the 10 texts, the left-hand graphs in Fig 4 show this conditional average divided by the mean interval length *R*_*Q*_ as a function of *n*, for *R*_*Q*_ = 2, 8, and 32. Without memory, the conditional average is identical to *R*_*Q*_. Due to the long-range memory, the conditional average after the short intervals (open circles) is well below 1, while it is well above 1 after the long intervals (full circles). The effect is enhanced when the segment length *n* is enlarged. The effect is also enhanced when the ranked intervals, as shown in the right-hand graphs in Fig 4, are divided into quarters and the conditional averages after the lowest quarter (open circles) and the largest quarter (full circles) are considered.

### Memory in the text when the words are substituted by ranks

Finally, we like to discuss if the memory quantified for the return intervals can be found directly in the text when each word is substituted by its rank. To this end, we first followed [7–10] and performed a fluctuation analysis. As in [10], we focus on the Detrended Fluctuation Analysis (here DFA2) [28] which in the last decade has become the standard method for detecting long-range memory in data sets. In DFA2, one considers a fluctuation function *F*(*s*) to detect the long-range memory. To obtain *F*(*s*), one divides the data of interest $\{y_{i}^{*}\},i=1,\ldots ,N$, into non-overlapping windows *μ* of lengths *s*. Then one focuses, in each segment *μ*, on the cumulated sum *Y*_*i*_ of the $\left\{y_{i}^{*}\right\}$, and determines the variance $F_{\mu }^{2}\left(s\right)$ of the *Y*_*i*_ around the best polynomial fit of order 2. After averaging $F_{\mu }^{2}\left(s\right)$ over all segments *μ* and taking the square root, one arrives at the desired fluctuation function *F*(*s*). One can show that in long-term persistent records where the autocorrelation function *C*(*s*) decays by a power law, $C(s)\;≅\;(1\;-\;\gamma )(1\;-\;\gamma /2)s^{-\gamma },\;0\;<\;\gamma \;<\;1$ , the fluctuation function increases by a power law,

$$F\left(s\right)∼s^{h},$$ (10)

where the exponent *h* can be associated with the Hurst exponent and is related to the correlation exponent *γ* by *h* = 1 − *γ*/2. For white-noise records, *h* = 1/2. Accordingly, an exponent *h* > 1/2 characterizes the long-term persistence in a record and can be easily obtained from a double logarithmic plot of *F* versus *s*, as long as the graph of *F*(*s*) represents a straight line in the double-logarithmic presentation.

**Fig 5:**
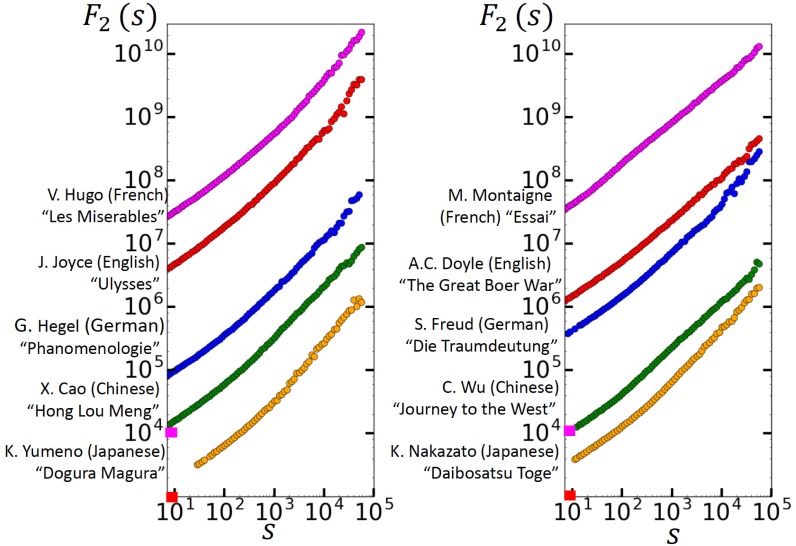
DFA2 fluctuation function *F*(*s*). The figure shows *F*(*s*) (in arbitrary units) for the 10 texts considered, where each word in a text has been substituted by its rank in the text.

Our results for the 10 texts considered (shown in Fig 5) confirmed the previous results [8–10] obtained for different texts. They show that the fluctuation functions in the double logarithmic presentation are not straight lines but show crossover behavior, from an exponent close to 0.5 at small scales to an exponent close to 1 at large scales. Shuffling of the texts leads to *F*(*s*) ∝ *s*^1/2^. Accordingly, the shape of *F*(*s*) clearly indicates some kind of long-range memory at large scales, but a specific law is difficult to derive from the behavior of *F*(*s*). It has been noticed in [25] that this kind of shape of *F*(*s*) characterizes records which exhibit both long-range memory and white noise (see the discussion above, Eq (8)). It has been suggested [25] that in this case, *F*(*s*) is not the appropriate function to look at. To accurately characterize the strength of white noise *and* long-range memory one has to study the autocorrelation function *C*(*s*) between the ranks of two words separated by *s* words. *C*(*s*) is defined as *C*_*Q*_(*s*), when *L*_*Q*_ is substituted by the length of the text, *r*_*i*_ by the rank of the *i*th word in the text and *R*_*Q*_ by the mean rank. It has been shown in [25] that the white noise only affects the prefactor in *C* but not the power-law decay.

**Fig 6:**
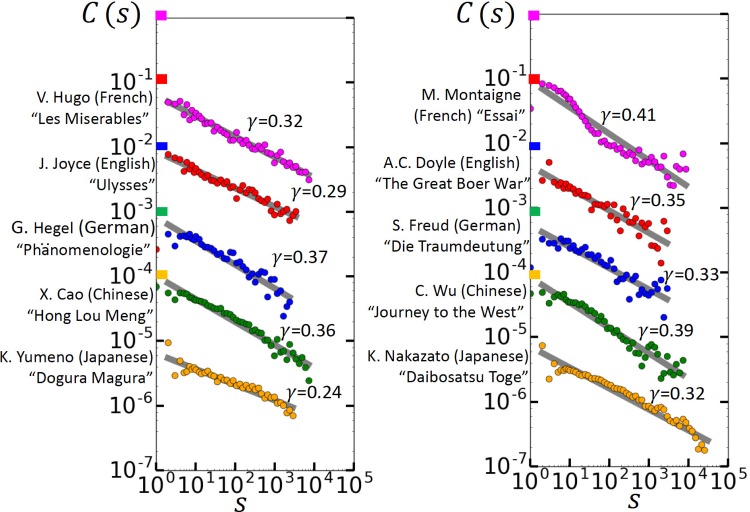
Long-range memory in the text when the words are substituted by their ranks. The figure shows the autocorrelation functions for the 10 texts considered. For transparency, we have multiplied *C*(*s*) for the 4 lower functions in both panels by 10^−1^,10^−2^, 10^−3^, and 10^−4^, respectively. Since autocorrelation functions are known to show strong finite-size effects [25], we considered only *s*-values up to (*N* − 1)/100. For *s* above 10, the data were binned logarithmically. The straight lines are the best linear fit to the data, for *s* ≥ 2. At *s* = 1, *C*(1) was negative for Les Miserables, Dogura Magura, Great Boer War and Daibosatsu Toge.

Fig 6 shows *C*(*s*) for the 10 texts considered. The figure shows that *C*(*s*), like *C*_*Q*_(*s*), decays by a clear power law in all texts, suggesting that the ranks of the words are long-range correlated. As a consequence, words with high (low) ranks are more likely to follow words with high (low) rank, and this in turn gives rise to the clustering of the rare words that we have discussed in the previous subsections. The exponents *γ* in *C*(*s*) are close to the exponents obtained for *C*_*Q*_(*s*). The figure also shows that the prefactor of *s*^−*γ*^ is well below the value (1 − *γ*)(1 − *γ*/2) for pure long-range correlated records, so we can conclude that in addition to long-range memory, there is a large fraction *a* of white noise in the rank representation of literary texts that can be estimated in a similar way as described above for the return intervals. Our estimations show that *a* is around 0.75: *a* = 0.76 for Les Miserables, 0.74 for Ulysses, 0.73 for Phänomenologie des Geistes, 0.71 for Hong Lou Meng, 0.77 for Dogura Magura, 0.71 for Essai, 0.79 for The Great Boer War, 0.78 for Die Traumdeutung, 0.72 for Journey to the West, and 0.75 for Daibosatsu Toge.

## Conclusions

In this article we considered 10 long literary texts from England/Ireland, France, Germany, China, and Japan and studied systematically the occurrence of the rare words in a text. We used techniques from the studies of extreme events which do not require a particular mapping of the words to numbers. We considered the fraction *N*_*Q*_/*N* of the rarest words in a literary text (that by definition have a rank above *Q*) and determined the return intervals between them, for fixed *Q*. Our major quantities of interest were the exceedance probability *S*_*Q*_(*r*) that the length of a return interval exceeds r, and the autocorrelation function of the intervals *C*_*Q*_(*s*). We found that for large threshold ranks *Q*, *S*_*Q*_(*r*) followed a perfect Weibull-function, while *C*_*Q*_(*s*) decays with *s* by perfect power-laws. When analyzing *C*_*Q*_(1) we found that the return intervals are not purely long-range correlated, but can be described as a superposition of white noise and a long-range correlated part. The long-range correlated part is responsible for the pronounced clustering of the rare words in a literary text.

We found that the same laws (Weibull functions for the exceedance probability and power-laws for the autocorrelation function of the return intervals) hold, with some variations in the parameters, for all languages considered, showing that the rhythm of a text quantified by the return intervals between the words, is surprisingly universal. This is particularly remarkable since the languages considered belong to different families and vary greatly [29, 30]. English, German, and French belong to the Indo-European family and use alphabetic writing systems, whereas Chinese belongs to the Sino-Tibetan family and uses a logosyllabary system. In contrast, Japanese adopts multiple writing systems, and its language family is unknown.

We consider the two laws as important “stylized” facts in languages that complement Zipf’s law. As Zipf’s law, both laws have been obtained empirically and lack a rigorous derivation by first principles. The results are universal in the sense that the same kind of functions describe the statistics of the return intervals, but the exponents are clearly not identical. For large thresholds (with *N*_*Q*_/*N* below 1/8), the exponents in the Weibull function vary between 0.68 and 0.86, and the exponents in the autocorrelation function vary between 0.14 and 0.48. In the texts considered, we found no indications that the exponents depend on the language considered.

We concentrated on the arrangements of the rare words in single-authored literary texts. For the quality of the analysis, we had to consider large texts, with more than 200,000 words. It would be interesting to see, if the arrangements of the rare words in single-authored texts is different from the arrangement in speeches. But since typical speeches consist only of few thousand words, a return-interval analysis as performed here may suffer from strong finite size effects.

Further extensive work is needed to see, to which extent the laws we find for single-author texts also hold for multi-author texts, and to which extent language engineering where the properties of rare words are crucial can benefit from our results. Preliminary work on 3 well recognized newspapers (see Fig D in S1 File) shows that the Weibull representation of *S*_*Q*_(*r*) is still valid, with exponents *β* slightly smaller than for the single-authored texts. Regarding *C*_*Q*_(*r*), the power-law decay is not as clear as for single authored texts.

## Supporting Information

S1 File The supporting information file includes Figs A-D and Table A, in addition to some additional mathematical explanation. (PDF)
