# Supplementary material for: Long-Range Memory in Literary Texts: On the Universal Clustering of the Rare Words
Source: PLoS One. 2016 Nov 28;11(11):e0164658. doi: 10.1371/journal.pone.0164658 (PMC5125566; doi:10.1371/journal.pone.0164658)
Supplement: S1 File — (PDF) [file pone.0164658.s001.pdf]

Supporting Information

Long-range memory in literary texts: On the universal clustering of the rare words

Kumiko Tanaka-Ishii[1‡], Armin Bunde[2‡*]

1 The University of Tokyo, Research Center for Advanced Science and Technology, Tokyo, 153-8904, Japan.
2 Universität Giessen,Institut für Theoretische Physik,Giessen,35392, Germany

‡These authors contributed equally to this work.
* armin.bunde@physik.uni-giessen.de

In this Supporting Information, we show first how the parameter b in the stretched exponential describing the exceedance probability can be determined. Then we present, in Figure A, the rank-frequency distribution of the 10 texts considered here and show in Figure B how the return period R_Q varies with rank Q in the texts. We demonstrate in Figure C that for shuffled texts the exceedance probability $S_Q(r)$ is a simple exponential and the autocorrelation function $C_Q(s)$ fluctuates around 0. The table in Table A summarizes all the statistics, obtained in the present study. Figure D shows $S_Q(r)$ and $C_Q(s)$ of Newspaper corpora in three languages.

The parameter b in the exceedance probability for large R_Q.

In the article we observed that the exceedance probability $S_Q(r)$ can be expressed by a stretched exponential $S_Q(r) = \exp(-b(r/R_Q)^\beta) \equiv \exp(-bx^\beta)$ for all texts considered. Here we show that for large R_Q where $x = r/R_Q$ can be (approximately) considered as continuous, the relation $b = [\int_0^\infty dx \exp(-x^\beta)]^\beta$ holds.

To see this, we note that in the continuous limit, the probability density distribution P_Q satisfies $P_Q = -R_Q dS_Q(x)/dx$, yielding $P_Q = (1/R_Q)bx^{\beta-1}\exp(-bx^\beta)$. By definition, $R_Q = \sum_{r=1}^\infty rP_Q(r)$. For $x = r/R_Q \ll 1$, the sum can be transformed into the integral $R_Q = R_Q \int_0^\infty dxbx^{\beta-1}\exp(-bx^\beta) = R_Q \int_0^\infty dx \exp(-bx^\beta)$, which then yields $b = [\int_0^\infty dx \exp(-x^\beta]^\beta$.

Zipf's law for the 10 texts considered

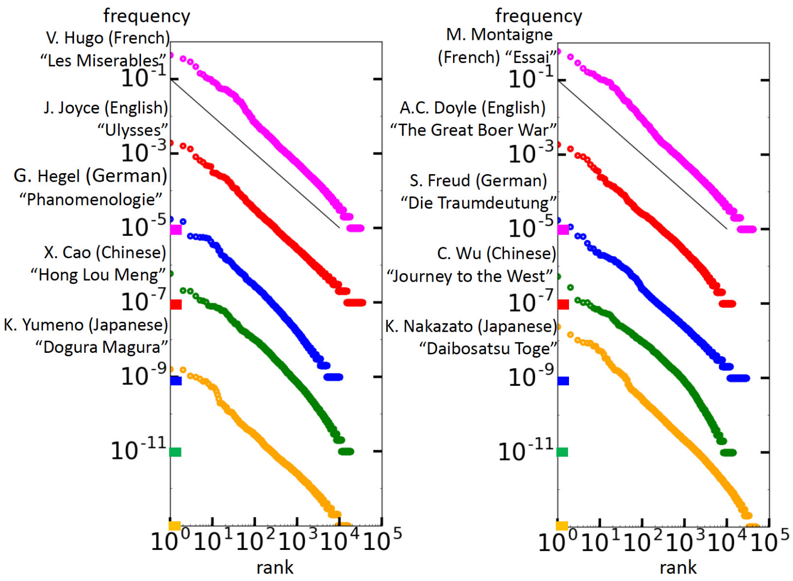

Figure A. Zipf's law for the 10 texts considered. Log-log plots of the rank-frequency distribution of the words in the 10 texts considered. The black line has slope -1 as suggested by Zipf's law. The figure demonstrates that Zipf's law is not rigorous but a reasonable approximation for the rank-frequency distribution in the considered texts.

Dependence of the return period R_Q on Q

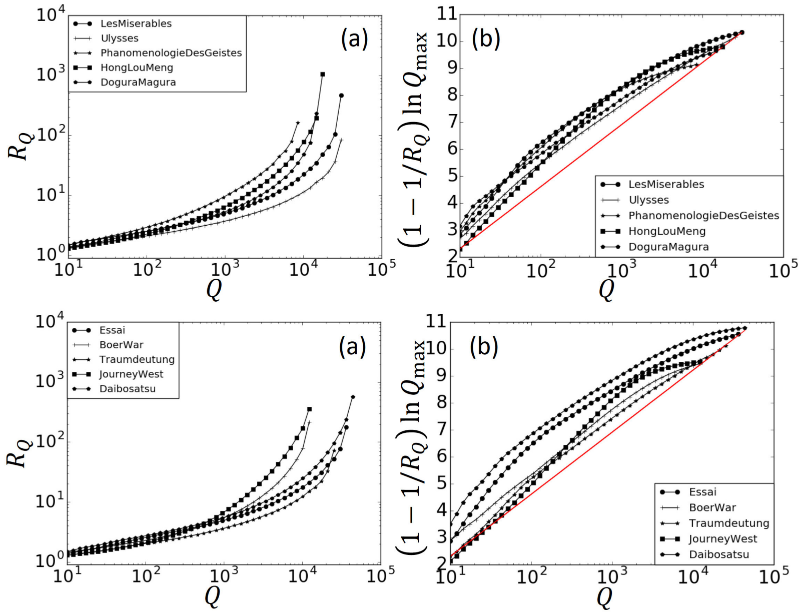

Figure B. **Dependence of the return period R_Q on Q.** (a) Log-log plots of R_Q as a function of Q (left shows plots for the first five texts and right shows those for the second five). From Zipf's law we would expect $R_Q = (1 - \ln Q / \ln Q_{max})^{-1}$, where Q_{max} is the maximum rank in the text. (b) Zipf's law suggests that $(1 - 1/R_Q) \ln Q_{max} = \ln Q$. Accordingly, when plotting $(1-1/R_Q) \ln Q_{max}$ versus $\ln Q$, deviations from the straight (red) line are due to the deviations from Zipf's law.

$S_Q(r)$ and $C_Q(s)$ in randomized texts

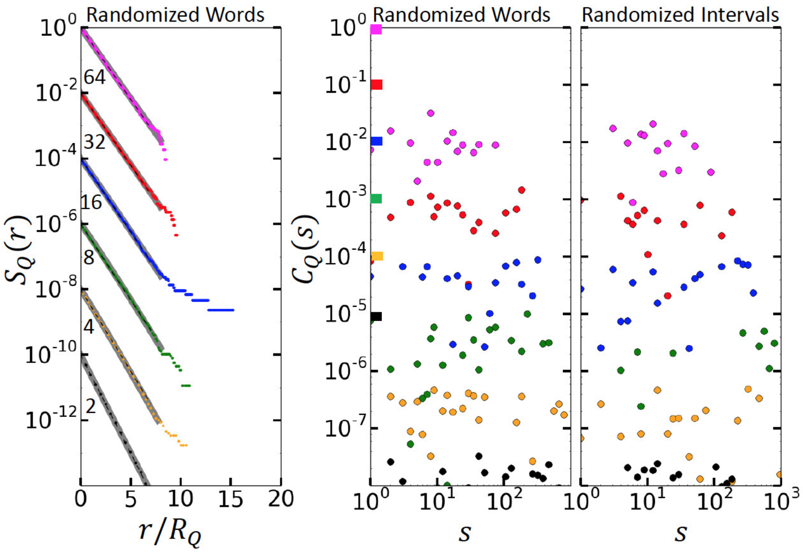

Figure C. **$S_Q(r)$ and $C_Q(s)$ in randomized texts.** The behavior of $S_Q(r)$ and $C_Q(s)$ for the randomized French text (*Les Miserables*). The left and middle figures show the results for the randomized order of words: $S_Q(r)$ coalesces with the theoretical result $S_Q(r) = (1 - 1/R_Q)^r \equiv \exp(-|\ln(1 - 1/R_Q)|r)$ (straight line), and $C_Q(s)$ fluctuates randomly around zero. Since the figure shows only the positive values, the points are sparse. The right figure shows $C_Q(s)$ calculated for the randomized intervals. By definition, $S_Q(r)$ of this data set is the same as that of the original text. As in the middle figure, $C_Q(s)$ fluctuates around zero.

Summary of parameters

Table A. Summary of parameters is presented in the following two pages. For fixed return period R_Q, the table shows (i) the values of the corresponding Q value and the number of words above Q, N_Q, (ii) the exponent β and the parameter b in $S_Q(r) = \exp(-b(r/R_Q)^\beta$, and (iii) the exponent γ and the parameter $C_Q(1)$ for the fitted autocorrelation function $C_Q(s) = C_Q(1)s^{-\gamma}$. From $C_Q(1)$ and γ, the fraction a of white noise can be estimated [24] $a = 1/[1 + \sqrt{C_Q(1)/(C_Q^{(0)}(1) - C_Q(1))}]$ where $C_Q^{(0)}(1) \cong (2-\gamma)(1-\gamma)/2$.

R_Q	2	4	8	16	32	64
**Les Miserables**						
Q	46	544	2731	7265	13799	21158
N_Q	344320	172789	86417	43202	21599	10799
β	1.0±0.020	0.91±0.0085	0.86±0.0017	0.76±0.00068	0.74±0.00043	0.73±0.00025
b	1.67	1.32	1.07	1.14	1.15	1.16
γ	0.56 ± 0.10	0.29 ± 0.013	0.32 ± 0.019	0.30 ± 0.016	0.30 ± 0.017	0.30 ± 0.031
$C_Q(1)$	0.04	0.09	0.15	0.15	0.18	0.20
a	0.72	0.71	0.62	0.60	0.58	0.59
**Ulysses**						
Q	80	1255	6114	14306	24187	29278
N_Q	162466	81418	40712	20356	10173	5087
β	1.0±0.035	0.76±0.0055	0.73±0.0016	0.70±0.0011	0.73±0.00089	0.76±0.00043
b	1.68	1.38	1.16	1.18	1.16	1.13
γ	—	0.24 ± 0.007	0.22 ± 0.010	0.24 ± 0.018	0.25 ± 0.016	0.22 ± 0.040
$C_Q(1)$	—	0.14	0.16	0.17	0.18	0.15
a	—	0.67	0.65	0.63	0.62	0.66
**Phänomenologie des Geistes**						
Q	31	215	765	1957	3975	6427
N_Q	109606	54962	27498	13759	6880	3440
β	0.94±0.022	0.82±0.011	0.85±0.0036	0.75±0.00081	0.68±0.00071	0.73±0.00054
b	1.59	1.37	1.07	1.14	1.19	1.16
γ	0.43 ± 0.044	0.29 ± 0.012	0.31 ± 0.020	0.32 ± 0.018	0.26 ± 0.034	0.27 ± 0.065
$C_Q(1)$	0.05	0.10	0.16	0.18	0.15	0.17
a	0.73	0.69	0.62	0.59	0.64	0.63
**Hong Lou Meng**						
Q	68	421	1389	3166	5849	9248
N_Q	351131	175746	87859	43927	21967	10984
β	0.94±0.0081	0.80±0.0018	0.78±0.00085	0.75±0.00048	0.74±0.00071	0.71±0.00027
b	1.42	1.28	1.12	1.14	1.14	1.17
γ	0.34 ± 0.015	0.38 ± 0.015	0.40 ± 0.016	0.36 ± 0.013	0.39 ± 0.030	0.37 ± 0.034
$C_Q(1)$	0.08	0.17	0.20	0.19	0.20	0.17
a	0.71	0.58	0.53	0.57	0.55	0.59
**Dogura Magura**						
Q	37	508	2073	4806	8202	11604
N_Q	136214	68471	34232	17119	8558	4280
β	1.00±0.034	0.85±0.0073	0.84±0.0016	0.76±0.00096	0.74±0.00067	0.74±0.00048
b	1.54	1.29	1.08	1.13	1.15	1.15
γ	0.11 ± 0.022	0.16 ± 0.018	0.28 ± 0.016	0.24 ± 0.019	0.28 ±0.036	0.21 ± 0.040
$C_Q(1)$	0.03	0.07	0.15	0.16	0.17	0.15
a	0.84	0.76	0.64	0.64	0.62	0.66

R_Q	2	4	8	16	32	64
**Essai**						
Q	43	539	3100	9040	18150	28388
N_Q	411227	205648	102824	51412	25702	12847
β	1.1±0.026	0.94±0.011	0.91±0.0023	0.82± 0.00054	0.78±0.00059	0.78±0.00035
b	1.58	1.33	1.04	1.10	1.12	1.12
γ	0.40±0.050	0.31 ± 0.012	0.32 ± 0.015	0.26 ± 0.015	0.17 ± 0.015	0.14 ± 0.021
$C_Q(1)$	0.028	0.068	0.10	0.13	0.13	0.13
a	0.80	0.73	0.68	0.67	0.68	0.69
**The Great Boer War**						
Q	56	549	1829	3928	6656	9513
N_Q	124463	62295	31165	15584	7790	3895
β	1.2±0.023	0.95±0.0097	0.94±0.0018	0.82±0.00081	0.80±0.00056	0.84±0.0010
b	1.49	1.29	1.03	1.09	1.11	1.08
γ	—	0.29 ± 0.032	0.32 ± 0.016	0.36 ± 0.035	0.40 ± 0.037	0.48 ± 0.16
$C_Q(1)$	—	0.05	0.11	0.13	0.13	0.14
a	—	0.78	0.68	0.64	0.62	0.57
**Die Traumdeutung**						
Q	96	1329	5736	13227	21049	24967
N_Q	125052	62628	31318	15637	7815	3897
β	1.2±0.013	0.92±0.0078	0.87±0.0023	0.80±0.00074	0.82±0.00048	0.86±0.00071
b	1.37	1.27	1.06	1.10	1.09	1.07
γ	—	0.29 ± 0.019	0.30 ± 0.018	0.33 ± 0.021	0.38 ± 0.069	0.43 ± 0.11
$C_Q(1)$	—	0.09	0.13	0.17	0.14	0.12
a	—	0.71	0.65	0.60	0.62	0.62
**Journey to the West**						
Q	89	478	1221	2361	4056	6294
N_Q	323934	162202	81113	40570	20280	10141
β	0.83±0.0036	0.78±0.0041	0.81±0.00099	0.78±0.00061	0.77±0.00055	0.81±0.00085
b	1.47	1.30	1.10	1.12	1.12	1.10
γ	0.33 ± 0.018	0.39 ± 0.014	0.40 ± 0.016	0.41 ± 0.021	0.38 ± 0.030	0.40 ± 0.040
$C_Q(1)$	0.10	0.14	0.15	0.16	0.14	0.13
a	0.68	0.61	0.60	0.59	0.61	0.62
**Daibosatsu Toge**						
Q	31	406	2243	6366	12848	20766
N_Q	1470548	737507	368861	184443	92224	46111
β	1.0±0.050	0.91±0.0057	0.90±0.0038	0.81±0.0016	0.76±0.00044	0.74±0.00015
b	1.43	1.28	1.05	1.10	1.13	1.15
γ	–	0.29 ± 0.017	0.36 ± 0.012	0.35 ± 0.011	0.34 ± 0.011	0.28 ±0.012
$C_Q(1)$	–	0.09	0.17	0.18	0.19	0.17
a	—	0.71	0.59	0.58	0.58	0.62

$S_Q(r)$ and $C_Q(s)$ of Newspaper Corpora

We also considered newspaper archives consisting of the Wall Street Journal (1987, 108 MB, N=22679512), 10 years of the People's Daily in Chinese (1995, 67 MB, N=19420852), and the Mainichi newspaper in Japanese (2000-2009, 1.3 GB, N=289032862). These corpora are thus multiple-author texts. The articles in each newspaper are aligned in chronological order and some rare words could therefore occur locally in the corpora. For the three languages, the exceedance probabilities and autocorrelation functions correspond to the left-hand, middle, and right-hand graphs, respectively, in Figure D.

For the exceedance probability, the Weibull function gives an excellent fit, especially for larger R_Q. In the case of the Chinese journal, the fit is perfect even for lower R_Q. Therefore, the Weibull characteristic underlying the distribution of intervals occurs independently of whether texts have single or multiple authors, although the values of β were smaller.

The autocorrelation function behaves differently from that for single-author texts. The plots have a tendency to present a convex alignment, indicating a shorter memory, leading to larger error bars than those in Fig 3. This can be understood, since newspaper articles are usually quite short, and hence the memory tends to decay faster than for single-author texts. A more detailed mathematical and experimental explanation of the difference between single- and multi-author texts remains for our future work.

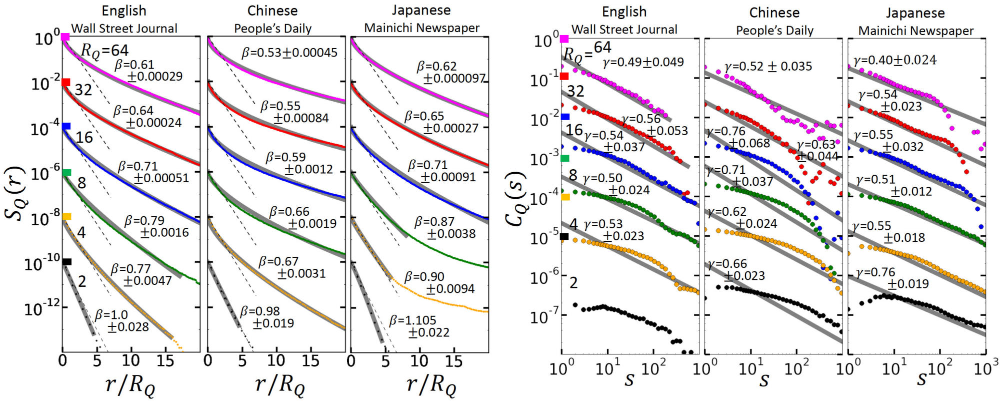

Figure D. Left block: Probability $S_Q(r)$, same as Fig 2, for large-scale English (Wall Street Journal, left), Chinese (People's Daily, middle), and Japanese (Mainichi, right) texts of Newspapers. Right block: Autocorrelation function $C_Q(s)$, same as Fig 3, for large-scale English (left), Chinese (middle) and Japanese (right) texts. For the autocorrelation function of $R_Q = 2$, the first data point was negative for the Wall Street Journal.
